# Predictors for functional decline after an injurious fall: a population-based cohort study

**DOI:** 10.1007/s40520-020-01747-1

**Published:** 2020-11-07

**Authors:** Stina Ek, Debora Rizzuto, Weili Xu, Amaia Calderón-Larrañaga, Anna-Karin Welmer

**Affiliations:** 1 Aging Research Center (ARC), Department of Neurobiology, Care Sciences and Society, Karolinska Institutet and Stockholm University, Stockholm, Sweden; 2 Unit of Epidemiology, the Institute of Environmental Medicine, Karolinska Institutet, Box 210, 171 77 Stockholm, Sweden; 3 Stockholm Gerontology Research Center, Stockholm, Sweden; 4 Allied Health Professionals, Function Area Occupational Therapy & Physiotherapy, Karolinska University Hospital, Stockholm, Sweden; 5 Division of Physiotherapy, Department of Neurobiology, Care Sciences and Society, Karolinska Institutet, Stockholm, Sweden

**Keywords:** Older adults, IADL, ADL, Disability trajectories, SNAC-K

## Abstract

**Background:**

The functional consequences of injurious falls are well known. However, studies of the factors that can modify trajectories of disability after an injury from a fall are scarce.

**Aims:**

We aimed to investigate whether sociodemographic and health-related factors may impact this association.

**Methods:**

The study population consisted of 1426 community-dwelling older adults (≥ 60 years) from the SNAC-K cohort study in Stockholm, Sweden. Functional status over 12 years of follow-up was assessed using the number of limitations in basic and instrumental activities of daily living. Sex, cohabitation status, physical activity, and self-rated health were assessed at baseline. Injurious falls were defined as falls requiring healthcare and were assessed over 3 years starting at baseline. Data were analyzed using linear-mixed effects models.

**Results:**

The fastest increase in the number of disabilities was observed in those who had endured an injurious fall and were living alone (*β* coefficient = 0.408; *p* < 0.001), been physically inactive (*β* coefficient = 0.587; *p* < 0.001), and had poor self-rated health (*β* coefficient = 0.514; *p* < 0.001). The negative impact of these factors was more pronounced among fallers compared to non-fallers.

**Discussion:**

Living alone, being physically inactive, and having poor self-rated health magnifies the negative effect of an injurious fall on functional status. Among individuals who endure an injurious fall, the heterogeneity in long-term functional status is substantial, depending on the individuals’ characteristics and behaviors.

**Conclusions:**

These findings emphasize the need for a person-centered approach in care provision and can guide secondary prevention within health care.

**Electronic supplementary material:**

The online version of this article (10.1007/s40520-020-01747-1) contains supplementary material, which is available to authorized users.

## Introduction

Injurious falls are the most common cause of hospitalization among older adults [1]. The consequences of such falls may be detrimental in terms of functional outcomes, leading to decreased quality of life for the individual and a high care burden for society [2]. Functional decline is most often defined by increased dependency in basic and instrumental activities of daily living [3–5]. Indeed, maintaining independence while aging is among the most important dimensions of health for older individuals themselves, and should, therefore, be prioritized [6]. A proper understanding of the long-term functional consequences of an injurious fall can guide care planning, rehabilitation, and resource allocation. While the functional consequences of an injurious fall are well established, possible factors that could impact this association are not well-understood. A few studies have shown that certain traits and characteristics can impact the trajectory of physical decline and dependence among older adults that have endured a fall [3, 7], but more studies are needed to establish which specific health-related and sociodemographic factors determine the disability trajectories of older adults that have endured an injurious fall.

Previous studies, including our research, suggest that men and women may differ in terms of risk factors for falls [8–10]. However, only a few studies have investigated whether recovery of functional status differs between men and women who survive a fall-injury hospitalization, and with inconsistent results [7, 11–13]. Living with someone has been shown to improve the course of disability after an injury [7], and findings from our research group have indicated that living alone is an important risk factor for injurious falls [14]. Both low physical activity and poor self-rated health are known risk factors for falls [15], and inactivity and poor health-related quality of life have shown to be consequences of an injury [2, 16].

In conclusion, there is a need to identify both modifiable and non-modifiable factors that may impact the development of functional decline after an injurious fall to determine which individuals are most at risk of not reaching full recovery. In this population-based study of older adults, we aimed to investigate the sociodemographic and health-related predictors of disability trajectories after an injurious fall.

## Methods

### Study population

We gathered data from the Swedish National Study on Aging and Care in Kungsholmen (SNAC-K) [17]. The population of Kungsholmen in central Stockholm, Sweden, was randomly sampled from 11 age cohorts (60, 66, 72, 78, 81, 84, 87, 90, 93, 96 and 99 +). In total, 4590 people were invited and 3363 participated (response rate 73.3%) in the baseline assessment (2001–2004). SNAC-K is an ongoing longitudinal study; people aged 60–72 are assessed every sixth year while people aged over 78 are assessed every third year. Participants in this study were followed for up to 12 years (2013–2016).

Of the total sample, we excluded participants who refused the use of their health register data (*n* = 62). Due to differences in post-acute care and to establish the exposure period, we also excluded those people who lived in nursing homes (*n* = 189) and those who had fallen after 3 years since baseline (*n* = 291). Furthermore, we excluded people who did not participate in any SNAC-K follow-up assessments (*n* = 1050) and those with missing data for any of the covariates or exposures (*n* = 251). The analytical sample consisted of 1426 individuals. The excluded participants were older, had lower levels of education, had a greater number of disabilities, were less physically active, and were more likely to be women than those in the analytical sample (*p* < 0.05).

SNAC-K was approved by the Regional Ethical Board in Stockholm and follows the Helsinki Declaration. All participants provided written informed consent and are free to leave the study whenever they wish to.

### Data assessment

In SNAC-K, trained nurses and physicians collected data based on structured interviews, clinical examinations and physical function tests. The full SNAC-K protocol is available at https://www.snac.org.

#### Outcome

Independence in activities of daily living (ADL) and instrumental ADL (IADL) were assessed through interviews. ADL included bathing, getting dressed, toileting, transferring, and eating. IADL included managing finances, using the telephone, grocery shopping, using public transportation, preparing meals, doing laundry, and cleaning. ADL and IADL items were combined into a disability score with a value ranging from 0 to 14 [18].

#### Exposures

An injurious fall was defined as a fall requiring inpatient or outpatient specialist care. The following discharge diagnoses from the International Classification of Diseases 10th revision (ICD-10) were used: W00, W01, W05–W10, and W17–W19. These were chosen to represent a fall caused by low force with no other person involved. The Swedish personal identification number (PIN) was used to link data from the National Patient Register and the Local Outpatient Register to each SNAC-K participant [19, 20]. In this study, we included injurious falls that occurred up to 3 years after the baseline examination as an exposure. Sex was derived from registers. Cohabitation status was assessed through interviews; people who were married or cohabiting were classified as living with someone, whereas unmarried, divorced, and widowed people were classified as living alone. Physical activity level was determined based on two questions from a self-administered questionnaire: (1) “Do you regularly engage in light exercise? (such as walking, shorter bicycling, and golf)” and (2) “Do you regularly engage in more intense exercise? (such as jogging or brisk long walks, heavy-duty gardening, high-intensity aerobics, skiing, swimming, and ball sports).” Both questions concerned the last 12 months and possible answers included: every day, several times per week, 2–3 times per month, less, and never. The two questions were combined in one dichotomous variable (inactive/active), based on current guidelines. Participants were considered active if they were engaged in light and/or intense exercise every day or several times per week and inactive for any of the other response options [21]. Self-rated health was assessed by a questionnaire including the question “In general, how would you describe your health?”; the answers “poor” and “fair” were categorized as “poor” while the answers “good”, “very good,” and “excellent” were categorized as “good”.

#### Covariates

Age was derived from registers. Education was assessed through interviews and refers to the highest level of education achieved (elementary, high school, or university). Cognition was assessed with the Mini-Mental State Examination (MMSE) test and cognitive impairment was defined as having a MMSE score of less than 28, because of the highly educated population studied [22]. Multimorbidity was defined as having two or more chronic diseases from a list developed by an interdisciplinairy team in a previous study by Calderón-Larrañaga et al. [23]. A disease was defined as chronic if it had a prolonged duration and either (1) led to residual disability or worsening quality of life, or (2) required long period of care, treatment, or rehabilitation. In SNAC-K, chronic conditions were determined based on a combination of clinical examination data, laboratory data, current drug use and health care register data. Data on survival was extracted from the Swedish Death Registers and was linked to the participants by the PIN. Survival status was measured throughout the whole follow-up and was categorized as alive or dead at the end of the study period.

### Statistical analysis

Each binary predictor—sex, cohabitation status, physical activity level, and self-rated health—was combined with injurious falls (falls/no falls) to create four different indicator variables, each with four mutually exclusive categories. Linear mixed-effects models with random effects for intercept and slope were used to examine the association between the indicator variables and the changes in disability score over time, resulting in four separate models. To measure the effect of the exposures on the average annual change in the number of disabilities, the interaction term between follow-up time (in years) and each of the four indicator variables was included as a fixed effect. All four models were adjusted for all other exposures, as well as age, education level, multimorbidity, and MMSE score. Survival was also included in the models initially but was omitted due to collinearity with other covariates. Non-linearity of follow-up time was tested but was not significant. Interactions between injurious falls and sex, living alone, inactivity, and self-rated health on disability were also tested separately.

#### Sensitivity analysis

To verify that the results were not driven by injurious falls that occurred before the baseline examination or by the severity of the injury (i.e. experiencing a fracture), we performed the following sensitivity analyses: (1) excluding individuals who had experienced an injurious fall within 3 years of the baseline assessment, and (2) defining injurious falls as falls that resulted in fractures. Finally, to take into accunt the missing data, multivariate imputation by chained equations (MICE) [24] was performed to obtain five imputed datasets. All variables included in the main analyses were used in the multiple imputation models.

### Results

Of the 1426 individuals in the study sample, 867 (60.8%) were women and the mean age was 69.3 (SD 8.5). Seventy-nine individuals (5.5%) experienced a fall between baseline and the three-year follow-up examination. Baseline characteristics of the analytical sample are presented in Table 1.

**Table 1:** Baseline characteristics of the analytical sample, *n* = 1426

Characteristics	Sample, *N* = 1426
Age, mean ± SD	69.3 ± 8.5
Women, *n* (%)	867 (60.8)
Education, *n* (%)	
Elementary	148 (10.4)
High School	681 (47.8)
University	597 (41.9)
Multimorbidity, *n* (%)	1157 (81.1)
MMSE <28, *n* (%)	94 (6.6)
Living alone, *n* (%)	643 (45.1)
Physically inactive, *n* (%)	255 (17.9)
Fair to poor self-rated health, *n* (%)	302 (21.2)
Fallers between baseline and 3 years, *n* (%)	79 (5.5)
Previous fallers (3 years prior to baseline), *n* (%)	66 (4.6)
Number of disabilities at baseline, mean ± SD	0.1 ± 0.6

Multimorbidity=2 or more diseases, *MMSE *mini mental state examination, physically inactive ≤ 1 activity/week

Data on baseline health status and the number of deaths and dropouts in the different study groups over the 12-year follow-up are presented in Table 2.

**Fig. 1:**
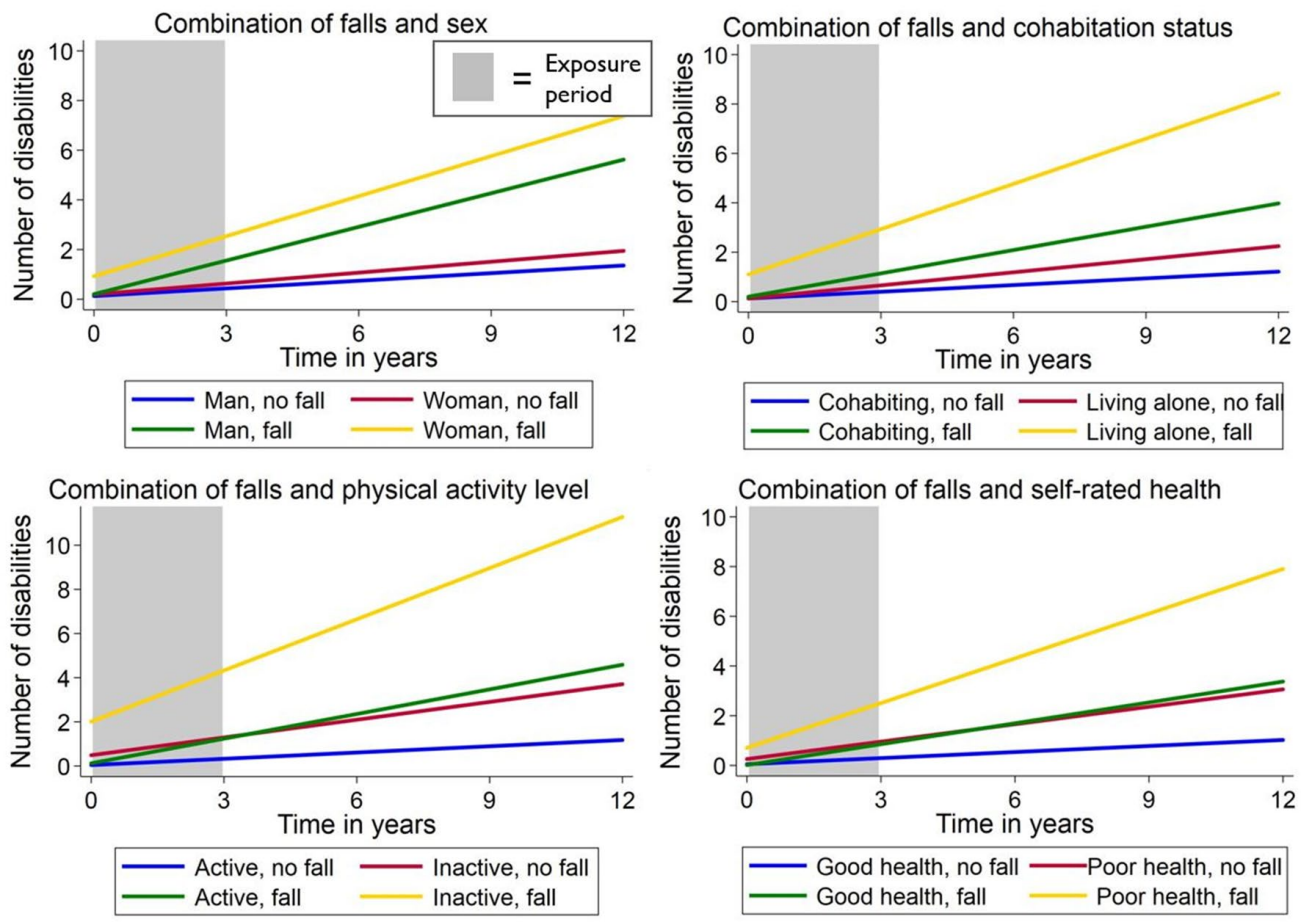
Predicted mean number of disabilities associated with injurious falls in combination with sex, cohabitation, physical activity level and self-rated health

Results from the linear mixed effects models indicated that women had more disabilities than men at baseline regardless of the future occurrence of a fall, although there were no sex differences in the disability trajectory over time (Table 3; Fig. 1). In terms of falls and cohabitation status, there were no differences in disability levels at baseline, but the number of disabilities increased fastest for fallers who lived alone (*β* coefficient = 0.408; *p* < 0.001). Physically inactive individuals had more disabilities than active individuals at baseline for both fallers and non-fallers, but the steepest disability trajectory was seen for inactive fallers (*β* coefficient = 0.587; *p* < 0.001). Individuals with poor self-rated health had higher levels of disability at baseline compared to those with good self-rated health, regardless of future falls, but the highest increase in disability was seen for fallers with poor self-rated health (*β* coefficient = 0.514; *p* < 0.001). In addition, non-fallers with poor self-rated health had an almost identical increase in disability as fallers with good self-rated health. The increase in the number of disabilities was higher among non-cohabiting vs. cohabiting fallers compared to non-cohabiting vs. cohabiting non-fallers (*β* coefficients 0.205 and 0.059, respectively), inactive vs. active fallers compared to inactive vs. active non-fallers (*β* coefficients 0.337 and 0.112, respectively), and fallers with poor vs. good self-rated health compared to non-fallers with poor vs. good self-rate health (*β* coefficients: 0.394 and 0.153, respectively) (Table 3; Fig. 1). The interaction between sex and falls on disability was not significant, but the interactions between falls and the other three exposures (living alone, inactivity and self-rated health) were (*p* < 0.05).

**Table 2:** Distribution of baseline multimorbidity, cognitive impairment, previous falls and number of deaths and dropouts during the 12-year follow-up, by the different groups of combinations of injurious falls with sex, cohabitation status, physical activity level and self-rated health

	*n*	Baseline multimorbidity (%)	Baseline cognitive impairment (%)	Previous falls (%)	Number of deaths at 12-year follow-up (%)	Number of dropouts at 12-year follow-up (%)
Sex						
Man, no fall	541	425 (78.6)	40 (7.4)	12 (2.2)	111 (20.5)	61 (11.3)
Woman, no fall	806	660 (81.9)	44 (5.5)	41 (5.1)	127 (15.8)	114 (14.2)
Man, fall	18	16 (88.9)	2 (11.1)	3 (16.7)	9 (50.0)	1 (5.6)
Woman, fall	61	56 (91.8)	8 (13.1)	10 (16.4)	19 (31.2)	8 (13.1)
Cohabitation						
Cohabiting, no fall	759	589 (77.6)	36 (4.7)	24 (3.2)	104 (13.7)	95 (12.5)
Alone, no fall	588	496 (84.4)	48 (8.2)	29 (4.9)	134 (22.8)	80 (13.6)
Cohabiting, fall	24	22 (91.7)	1 (4.2)	2 (8.3)	7 (29.2)	2 (8.3)
Alone, fall	55	50 (90.9)	9 (16.4)	11 (20.0)	21 (38.2)	7 (12.7)
Physical activity						
Active, no fall	1113	895 (80.4)	66 (5.9)	41 (3.7)	192 (17.3)	136 (12.2)
Inactive, no fall	234	190 (81.2)	18 (7.7)	12 (3.1)	46 (19.8)	39 (16.8)
Active, fall	58	51 (87.9)	7 (12.1)	7 (12.1)	16 (27.6)	7 (12.1)
Inactive, fall	21	21 (100.0)	3 (14.3)	6 (28.6)	12 (57.1)	2 (9.5)
Self-rated health						
Good, no fall	1083	832 (76.8)	61 (5.6)	40 (3.7)	153 (14.1)	141 (13.0)
Poor, no fall	264	253 (95.8)	23 (8.7)	13 (4.9)	85 (32.3)	34 (12.9)
Good, fall	41	36 (87.8)	7 (17.1)	7 (17.1)	9 (22.0)	5 (12.2)
Poor, fall	38	36 (94.7)	3 (7.9)	6 (15.8)	19 (50.0)	4 (10.5)

**Table 3:** *β* coefficient and 95% confidence intervals (CI) for the association between injurious falls in combination with sex, cohabitation, physical activity level and self-rated health and changes in disability over 12 years

	*n*	Baseline, *β*	(95% CI)	*p*	Annual change, *β*	95% CI	*p*
Sex							
Man, no fall	541	Ref			Ref		
Woman, no fall	806	0.105	0.000–0.209	**0.048**	0.014	− 0.017 to 0.047	0.365
Man, fall	18	0.109	− 0.318 to 0.536	0.616	0.341	0.200–0.482	** < 0.001**
Woman, fall	61	0.310	0.066–0.554	**0.013**	0.324	0.246–0.402	** < 0.001**
Cohabitation							
Cohabiting, no fall	759	Ref			Ref		
Alone, no fall	588	− 0.068	− 0.172 to 0.037	0.203	0.059	0.027–0.090	** < 0.001**
Cohabiting, fall	24	− 0.062	− 0.434 to 0.310	0.742	0.203	0.086–0.320	** < 0.001**
Alone, fall	55	0.229	− 0.023 to 0.480	0.075	0.408	0.328–0.489	** < 0.001**
Physical activity							
Active, no fall	1113	Ref			Ref		
Inactive, no fall	234	0.187	0.058–0.316	**0.005**	0.112	0.071–0.152	** < 0.001**
Active, fall	58	− 0.082	− 0.319 to 0.154	0.495	0.250	0.174–0.326	** < 0.001**
Inactive, fall	21	1.123	0.731–1.517	** < 0.001**	0.587	0.458–0.717	** < 0.001**
Self-rated health							
Good, no fall	1083	Ref			Ref		
Poor, no fall	264	0.194	0.067–0.321	**0.003**	0.153	0.115–0.192	** < 0.001**
Good, fall	41	− 0.034	− 0.314 to 0.245	0.811	0.120	0.112–0.288	** < 0.001**
Poor, fall	38	0.663	0.358–0.959	** < 0.001**	0.514	0.420–0.609	** < 0.001**

Controlled for age, education, multimorbidity, MMSE and the other exposure variables (sex, living alone, physical activity level and self-reported health) when applicable. Significant *p *values on a 95% confidence interval level in bold

#### Sensitivity analyses

The analyses excluding previous fallers and when only considering injurious falls leading to fractures yielded similar results to the original analyses, suggesting that neither injurious falls that occurred before the baseline examination nor the severity of the injury (i.e. experiencing a fracture) was driving the results (Supplementary Tables 1 and 2). Finally, results from the imputed datasets were similar to those from the complete case analyses (Supplementary Table 3).

## Discussion

In this population-based longitudinal study of older adults, we found that living alone, being physically inactive, and having a poor self-rated health predicted steeper declines in disability over a 12-year period, and these differences were even greater among fallers compared to non-fallers.

The predictors explored in this study can be categorized into modifiable and non-modifiable factors. Modifiable factors, such as physical activity, can be targeted for either primary prevention (i.e., prevention of falls) as well tertiary prevention (i.e., prevention of the long-lasting complications due to falls), by encouraging increased physical activity levels in the general older population [25]. In addition, the number of disabilities at baseline already differed between future fallers and non-fallers, by physical activity level and self-rated health. This could indicate that the injurious fall is a consequence rather than a cause of the increased disability level. Findings from Gill et al. might support this: They demonstrated that the course of disability before a fall is highly interrelated with the course of disability after a fall [26].

In this study, we did not find an association between sex and disability trajectories after an injurious fall. Previous studies on this have shown inconsistent results: one study concluded that women have a better prognosis [12], another study reported that men regain higher levels of physical function [11], and yet another showed no significant differences between men and women [13]. In this study, we controlled for health-related factors that are known to differ between men and women (e.g., age, education, cognition and multimorbidity), which may explain the non-significant result reported here. In addition, as shown in Table 2, men had a lower rate of survival in this study, which may result in a selection bias for the men.

In line with Bell et al.’s results, we showed that individuals living with a spouse had a better functional trajectory after an injury [7]. Social support has shown to be important for successful recovery after an injury [27], and the mechanisms behind this could relate not only to physical and emotional support but also adherence to treatment. DiMatteo et al. have shown in a meta-analysis that adherence to treatment is associated with social support [28].

Our results showed that being physically inactive prior to an injurious fall predicted a worse disability trajectory. Physical activity interventions with the aim to both reduce the risk of falls and to enhance the activity level after a fall have shown positive results [29, 30], confirming that such interventions might be an optimal primary and also tertiary prevention strategy to decrease the burden of falls among older adults.

Our results indicate that self-rated health prior to a fall is strongly associated with the course of disability, even after controlling for objective health-related factors such as age, multimorbidity and cognitive status. This is in line with the findings of Brenowitz et al. who showed that low self-rated health predicted decline in physical function [31]. Self-rated health is a comprehensive concept that may reflect not only an individual’s spontaneous view of their current health but also their health-related goals [32]. It appears that an individual’s subjective perception of health can alter the functional outcome after an adverse event, regardless of their objective health. This might reflect an individual’s resilience, including their health perceptions and expectations. Indeed, Ayalon et al. showed that satisfaction with aging can be protective against falls and suggested psychosocial interventions to decrease the risk of falling [33].

As for practical applications, the results from this study can be viewed as a basis for detecting individuals who are especially vulnerable to a worse prognosis after an injurious fall, with a possible underlying frailty phenotype [34]. Our findings also provide guidance regarding the maintenance of function and independence for individuals who fall through tertiary prevention. This is in line with the concept of physical resilience, which emphasizes the need to enhance and focus on protective factors instead of risk factors [35].

The proportion of fallers that died or dropped out from the study before the end of the follow-up was significantly higher than that of non-fallers, indicating that primary prevention measures to decrease falls among older adults is an essential tool to increase not only life-expectancy but also disability-free life years [36].

Strengths of this study include the use of population-based data including a number of objective health measurements as well as the linkage of this data to an almost full-coverage health care register, minimizing the risk of recall bias. Furthermore, the long follow-up period in this study adds novel information to research in this field. However, it is possible that factors impacting functional decline changed between waves of data collection in SNAC-K, and this was not considered in our study. There are also other limitations to consider. Some of the variables used in this study—although validated and commonly used in clinic—are rather crude measures lacking in detail; this includes the MMSE, the aggregate measure of ADL/IADL, and the definition of multimorbidity as ≥ 2 diseases. Additionally, mortality can be a competing risk when following older individuals for such a long time, especially among the subgroup of fallers. That said, survival status was omitted from the models due to collinearity, and death did not affect the disability trajectories beyond the other covariates. Finally, the study population comes from a wealthy area and may not be representative of the general aging population. Thus, our results need to be confirmed in other cohorts.

In conclusion, this study suggests that it is possible to predict functional decline in older adults after a fall using sociodemographic and health-related factors. After controlling for potential confounders, we found that living alone, being physically inactive, and having a poor self-rated health predicted a steeper decline in disability, and these differences were even more accentuated among fallers compared to non-fallers. This indicates that even among individuals who endured an injurious fall there is substantial heterogeneity in long-term functional status depending on individual characteristics and behaviors, emphasizing the need for a person-centered approach in care provision. In addition, some of these characteristics, such as physical activity level, are modifiable and can be targeted for primary as well as tertiary prevention of falls.

## Electronic supplementary material

Below is the link to the electronic supplementary material.

Supplementary material 1 (DOCX 32 kb)
