# Supplementary material for: Predictors for functional decline after an injurious fall: a population-based cohort study
Source: Aging Clin Exp Res. 2020 Nov 7;33(8):2183–90. doi: 10.1007/s40520-020-01747-1 (PMC8302494; doi:10.1007/s40520-020-01747-1)
Supplement: Supplementary file 1 — Supplementary material 1 (DOCX 32 kb) [file 40520_2020_1747_MOESM1_ESM.docx]

# Supplementary material

**Supplementary table 1**. β coefficient and 95% confidence intervals (CI) for the associatoin between injurious falls resulting in a fracture in combination with sex, cohabitation, physical activity level and self-rated health and changes in disability over 12, n=1,370.

|  | **Baseline, β** | **(95% CI)** | **p** | **Annual change, β** | **95% CI** | **p** |
| --- | --- | --- | --- | --- | --- | --- |
| **Sex** |  |  |  |  |  |  |
| Man, no fall | Ref. |  |  | Ref. |  |  |
| Woman, no fall | 0.113 | (0.011-0.215) | **0.030** | 0.018 | (-0.013-0.049) | 0.259 |
| Man, fall | 0.138 | (-0.479-0.755) | 0.660 | 0.265 | (0.069-0.461) | **0.008** |
| Woman, fall | 0.569 | (0.276-0.863) | **<0.001** | 0.254 | (0.159-0.350) | **<0.001** |
| **Cohabitation** |  |  |  |  |  |  |
| Cohabiting, no fall | Ref. |  |  | Ref. |  |  |
| Alone, no fall | -0.075 | (-0.177-0.027) | 0.149 | 0.059 | (0.028-0.090) | **<0.001** |
| Cohabiting, fall | 0.122 | (-0.380-0.623) | 0.634 | 0.133 | (-0.024-0.290) | 0.098 |
| Alone, fall | 0.431 | (0.125-0.736) | **0.006** | 0.324 | (0.224-0.425) | **<0.001** |
| **Physical activity** |  |  |  |  |  |  |
| Active, no fall | Ref. |  |  | Ref. |  |  |
| Inactive, no fall | 0.193 | (0.066-0.319) | **0.003** | 0.115 | (0.075-0.155) | **<0.001** |
| Active, fall | 0.192 | (-0.100-0.484) | 0.197 | 0.158 | (0.064-0.252) | **<0.001** |
| Inactive, fall | 1.294 | (0.757-1.831) | **<0.001** | 0.640 | (0.464-0.816) | **<0.001** |
| **Self-rated health** |  |  |  |  |  |  |
| Good, no fall | Ref. |  |  | Ref. |  |  |
| Poor, no fall | 0.203 | (0.077-0.328) | **0.002** | 0.159 | (0.121-0.197) | **<0.001** |
| Good, fall | 0.256 | (-0.075-0.586) | 0.129 | 0.171 | (0.067-0.275) | **<0.001** |
| Poor, fall | 0.862 | (0.449-1.273) | **<0.001** | 0.438 | (0.306-0.571) | **<0.001** |

Controlled for age, education, multimorbidity, MMSE and the other exposure variables (sex, living alone, physical activity level and self-reported health) when applicable. Significant p-values on a 95% confidence interval level in **bold**.

**Supplementary table 2**. β coefficient and 95% confidence intervals (CI) for the associatoin between injurious falls in combination with sex, cohabitation, physical activity level and self-rated health and changes in disability over 12 years in an subsample of first time fallers, n=1,360

|  | **Baseline, β** | **(95% CI)** | **p** | **Annual change, β** | **95% CI** | **p** |
| --- | --- | --- | --- | --- | --- | --- |
| **Sex** |  |  |  |  |  |  |
| Man, no fall | Ref. |  |  | Ref. |  |  |
| Woman, no fall | 0.085 | (-0.019-0.189) | 0.109 | 0.008 | (-0.024-0.391) | 0.642 |
| Man, fall | 0.088 | (-0.371-0.547) | 0.708 | 0.300 | (0.149-0.450) | **<0.001** |
| Woman, fall | 0.337 | (0.077-0.598) | **0.011** | 0.320 | (0.237-0.403) | **<0.001** |
| **Cohabitation** |  |  |  |  |  |  |
| Cohabiting, no fall | Ref. |  |  | Ref. |  |  |
| Alone, no fall | -0.070 | (-0.174-0.034) | 0.189 | 0.048 | (0.017-0.080) | **0.002** |
| Cohabiting, fall | -0.065 | (-0.447-0.317) | 0.740 | 0.199 | (0.080-0.317) | **<0.001** |
| Alone, fall | 0.283 | (0.009-0.559) | **0.043** | 0.403 | (0.009-0.559) | **<0.001** |
| **Physical activity** |  |  |  |  |  |  |
| Active, no fall | Ref. |  |  | Ref. |  |  |
| Inactive, no fall | 0.192 | (0.062-0.322) | **0.004** | 0.095 | (0.054-0.136) | **<0.001** |
| Active, fall | -0.052 | (-0.301-0.197) | 0.682 | 0.252 | (0.173-0.331) | **<0.001** |
| Inactive, fall | 1.291 | (0.830-1.752) | **<0.001** | 0.598 | (0.444-0.752) | **<0.001** |
| **Self-rated health** |  |  |  |  |  |  |
| Good, no fall | Ref. |  |  | Ref. |  |  |
| Poor, no fall | 0.180 | (0.052-0.307) | **0.006** | 0.138 | (0.099-0.176) | **<0.001** |
| Good, fall | -0.008 | (-0.312-0.296) | 0.959 | 0.195 | (0.101-0.290) | **<0.001** |
| Poor, fall | 0.676 | (0.361-0.991) | **<0.001** | 0.495 | (0.393-0.597) | **<0.001** |

Controlled for age, education, multimorbidity, MMSE and the other exposure variables (sex, living alone, physical activity level and self-reported health) when applicable. Significant p-values on a 95% confidence interval level in **bold**.

**Supplementary table 3**. β coefficient and 95% confidence intervals (CI) for the associatoin between injurious falls in combination with sex, cohabitation, physical activity level and self-rated health and changes in disability over 12 years in an imputed full sample of 1,677 individuals.

|  | **Baseline, β** | **(95% CI)** | **p** | **Annual change, β** | **95% CI** | **p** |
| --- | --- | --- | --- | --- | --- | --- |
| **Sex** |  |  |  |  |  |  |
| Man, no fall | Ref. |  |  | Ref. |  |  |
| Woman, no fall | 0.117 | (0.003-0.231) | **0.044** | 0.045 | (0.012-0.078) | **0.008** |
| Man, fall | 0.091 | (-0.333-0.514 | 0.675 | 0.346 | (0.212-0.480) | **<0.001** |
| Woman, fall | 0.772 | (0.517-1.026) | **<0.001** | 0.439 | (0.363-0.516) | **<0.001** |
| **Cohabitation** |  |  |  |  |  |  |
| Cohabiting, no fall | Ref. |  |  | Ref. |  |  |
| Alone, no fall | -0.089 | (-0.202-0.024) | 0.124 | 0.085 | (0.052-0.117) | **<0.001** |
| Cohabiting, fall | -0.005 | (-0.394-0.383) | 0.978 | 0.221 | (0.104-0.338) | **<0.001** |
| Alone, fall | 0.637 | (0.379-0.896) | **<0.001** | 0.513 | (0.435-0.591) | **<0.001** |
| **Physical activity** |  |  |  |  |  |  |
| Active, no fall | Ref. |  |  | Ref. |  |  |
| Inactive, no fall | 0.409 | (0.271-0.547) | **<0.001** | 0.173 | (0.133-0.214) | **<0.001** |
| Active, fall | 0.045 | (-0.219-0.309) | 0.738 | 0.277 | (0.198-0.356) | **<0.001** |
| Inactive, fall | 1.865 | (1.521-2.210) | **<0.001** | 0.678 | (0.572-0.784) | **<0.001** |
| **Self-rated health** |  |  |  |  |  |  |
| Good, no fall | Ref. |  |  | Ref. |  |  |
| Poor, no fall | 0.124 | (-0.026-0.275) | 0.105 | 0.170 | (0.113-0.227) | **<0.001** |
| Good, fall | 0.177 | (-0.141-0.495) | 0.273 | 0.260 | (0.161-0.358) | **<0.001** |
| Poor, fall | 1.007 | (0.686-1.329) | **<0.001** | 0.592 | (0.494-0.689) | **<0.001** |

Controlled for age, education, multimorbidity, MMSE and the other exposure variables (sex, living alone, physical activity level and self-reported health) when applicable. Significant p-values on a 95% confidence interval level in **bold**.
